# Neuroticism Delays Detection of Facial Expressions

**DOI:** 10.1371/journal.pone.0153400

**Published:** 2016-04-13

**Authors:** Reiko Sawada, Wataru Sato, Shota Uono, Takanori Kochiyama, Yasutaka Kubota, Sayaka Yoshimura, Motomi Toichi

**Affiliations:** 1 Primate Research Institute, Kyoto University, Inuyama, Aichi, Japan; 2 Graduate School of Medicine, Kyoto University, Kyoto, Japan; 3 Brain Activity Imaging Center, Advanced Telecommunications Research Institute International, Soraku-gun, Kyoto, Japan; 4 Health and Medical Services Center, Shiga University, Otsu, Shiga, Japan; University of Tuebingen Medical School, GERMANY

## Abstract

The rapid detection of emotional signals from facial expressions is fundamental for human social interaction. The personality factor of neuroticism modulates the processing of various types of emotional facial expressions; however, its effect on the detection of emotional facial expressions remains unclear. In this study, participants with high- and low-neuroticism scores performed a visual search task to detect normal expressions of anger and happiness, and their anti-expressions within a crowd of neutral expressions. Anti-expressions contained an amount of visual changes equivalent to those found in normal expressions compared to neutral expressions, but they were usually recognized as neutral expressions. Subjective emotional ratings in response to each facial expression stimulus were also obtained. Participants with high-neuroticism showed an overall delay in the detection of target facial expressions compared to participants with low-neuroticism. Additionally, the high-neuroticism group showed higher levels of arousal to facial expressions compared to the low-neuroticism group. These data suggest that neuroticism modulates the detection of emotional facial expressions in healthy participants; high levels of neuroticism delay overall detection of facial expressions and enhance emotional arousal in response to facial expressions.

## Introduction

Communication via facial expressions is a fundamental component of human social interaction. The ability to immediately detect emotional signals from facial expressions enables the receiver to interpret emotional states, anticipate the subsequent actions of the sender, and choose the appropriate response[1].

The processing of emotional facial expressions is modulated by personality; for example, neuroticism, a dimension of the five-factor model of personality [2, 3] has been found to play a role in this regard. Neuroticism is characterized by the tendency to be anxious, nervous, and hostile [3, 4]. Neuroticism is also considered to be a risk predictor for depression [5]. It has been suggested that neuroticism consists of two critical sub-factors [6]: (i) “withdrawal” which is a tendency characterized by general avoidance, irrespective of emotional valence; and (ii) “volatility,” which is a high sensitivity to negative signals from the environment.

Consistent with such notions, previous studies reported two characteristics in response to facial expressions, avoidance of facial expressions and sensitivity to negative emotions expressed in facial expressions. Some studies have shown that individuals with high-neuroticism tend to avoid emotional facial expressions. For example, scores on neuroticism were negatively related to accuracy in recognizing facial emotions [7]. High-neuroticism participants looked at the eyes of emotional facial expressions for shorter durations [8] or had less gaze maintenance to the eyes of fearful faces [9] than low-neuroticism participants. On the other hand, other scholarly work has provided evidence that individuals with high-neuroticism levels showed higher sensitivity to facial expressions depicting negative emotions compared to those with low-neuroticism. For example, high-neuroticism was associated with a higher emotional response to social interactions [10] and there was a positive relationship between scores on neuroticism and performance in recognizing emotional signals in fearful faces [11]. Overall, previous studies have indicated that neuroticism as a personality factor modulates the processing of facial expressions through withdrawal from potential emotions or a sensitive response to negative emotional signals.

Rapid detection of emotional signals is a critical component in processing facial expressions, allowing for immediate and appropriate responses to other people and the surrounding environment [12]. Previous studies using a visual search paradigm have shown a shorter reaction time (RT) for detecting emotional facial expressions (e.g., angry, happy) than neutral expressions [12–14]. This rapid detection of facial expressions has been attributed to the emotional significance of the expressions rather than to their visual features [14]. One study reported that neuroticism in participants modulates the rapid detection of facial expressions [15]. This study found that participants with high levels of neuroticism had longer RTs for detecting facial targets in the visual search paradigm. Although the researchers found a face-specific delay for detecting targets in the high-neuroticism participants compared to low-neuroticism participants, they did not compare the detection speed between emotional and neutral facial expressions. Therefore, it remains undetermined whether participants with high- and low-neuroticism levels could differ in their detection of emotional versus neutral expressions. To further assess the effects of neuroticism on rapid processing of facial expressions, we have investigated the modulatory effects of neuroticism on detecting emotional and neutral facial expressions.

Previous studies have shown that faster detection of facial expressions is associated with higher subjective arousal ratings [14, 16, 17] and lower valence ratings [16] in healthy participants. However, no extant study has tested the modulatory effects of neuroticism on this relationship. Based on previous studies showing enhanced emotional sensitivity to negative signals in high-neuroticism participants [11], it is possible that neuroticism can moderate the relationship between subjective emotional ratings and RTs.

**Fig 1:**
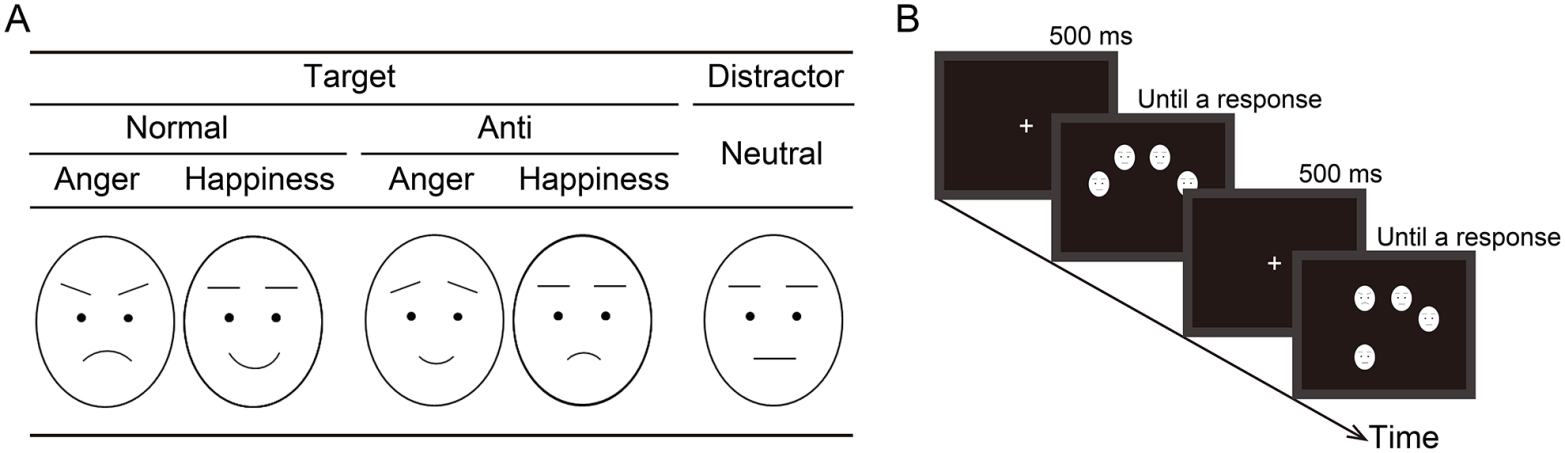
Schematic illustrations of stimuli (A) and presentation of stimuli in the visual search task (B). Actual stimuli were photographs of faces (see Fig 1 in Sato and Yoshikawa [14]).

In this study, participants with high- and low-neuroticism scores performed a visual search task, in which they were presented with photographs of normal expressions of anger and happiness and their “anti-expressions” [18] within crowds of neutral expressions (Fig 1). Anti-expressions were control stimuli created by morphing neutral expressions with the equivalent visual changes as normal emotional expressions compared to neutral expressions in the opposite direction [18]; anti-expressions were most frequently labeled or categorized as neutral expressions as opposed to other emotions (e.g., anger, happiness) [17–19]. Moreover, to investigate the emotional processes in response to facial expressions, the participants provided subjective evaluations of arousal and valence [20]. This method allowed us to determine whether the differences between high- and low-neuroticism participants in detection performance were attributable to emotional significance or to basic visual processing. The degree of familiarity and naturalness of the facial stimuli were assessed as possible confounding factors [21]. Based on their tendency to withdraw from facial expressions [8, 15], we predicted that high-neuroticism participants, relative to low-neuroticism participants, would show slower detection of facial targets over both emotional and neutral expressions. Additionally, given the association of neuroticism and sensitivity to negative emotional signals [10, 11], we also predicted that the high-neuroticism group would show a more robust relationship between subjective negative feelings and RTs of detecting facial expressions compared to the low-neuroticism group.

## Materials and Methods

### Ethics Statement

This study was part of a broad research project on the mind-brain relationship approved by the Ethics Committee of the Primate Research Institute at Kyoto University, and was conducted in accordance with the Declaration of Helsinki. All participants gave written informed consent after being provided with an explanation of the experimental procedure.

### Participants

A total of 74 participants (35 females and 39 males, mean ±*SD* age = 23.2 ± 4.1 years) completed the Japanese version of the NEO Five-Factor Inventory (NEO-FFI) [22]. The NEO-FFI, a 60-item self-rating questionnaire based on the five factor model [2], was used to measure scores of neuroticism. Participants with neuroticism scores in the top and bottom quartiles were defined as the high (*N* = 19, 12 females, mean ± *SD* age = 23.5 ± 5.5 years, mean ± *SD* neuroticism score = 38.6 ± 1.1) and low (*N* = 19, 9 females, mean ± *SD* age = 23.2 ± 3.7 years, mean ± *SD* neuroticism score = 19.7 ± 0.7) neuroticism groups, respectively. Big-five scores for the high- and low-neuroticism groups are shown in the Supplemental information (S1 Table). All participants were right-handed as assessed by the Edinburgh Handedness Inventory [23] and had normal or corrected-to-normal visual acuity.

### Stimuli

Normal and anti-expressions of angry and happy faces were used as target stimuli, and neutral expressions were used as distractor stimuli. This stimulus set was the same one used in previous studies that implemented the visual search paradigm [14, 16, 17]. The schematic images of the stimuli are shown in Fig 1A, although the actual stimuli were photographs of faces (see, Fig 1 in Sato and Yoshikawa [14]). Each individual face subtended a visual angle of 1.8° horizontally and 2.5° vertically. Normal expressions (i.e., unchanged) were grayscale photographs of a female (PF) and a male (PE) model with angry, happy, and neutral expressions drawn from a database of facial expressions [24]. The models were not familiar to any of the participants. Bared teeth and hair were not shown in any of the facial expressions. Anti-expressions were created from normal expressions using computer-morphing software (FUTON System, ATR-Promotions) [18]. The researchers identified the coordinates of 79 facial feature points of normal emotional (angry, happy) and neutral expressions manually and calculated the differences between the points of emotional and neutral facial expressions. Then, they determined the positions of the feature points for the anti-expressions by moving each point of the neutral expressions by the same distance as for normal expressions but in the opposite direction. Thus, relative to neutral expressions, anti-expressions contained equivalent visual changes as normal emotional expressions of anger and happiness; however, the anti-expressions were recognized most frequently as neutral expressions over the other emotions (e.g., anger, happiness) [17–19].

Stimuli were presented in eight positions separated by 45° and arranged in a circle (10.0° × 10.0°). Facial stimuli occupied four of the eight positions; half were presented to the left side and half to the right side. Each combination of the four positions was presented an equal number of times. In the target-present trials, the position of the target stimulus was randomly chosen, but they were presented to the left and right sides an equal number of times. In the target-absent trials, all four faces were neutral expressions.

### Apparatus

The experiment was conducted in a soundproofed room (Science Cabin, Takahashi Kensetsu). The stimulus presentation was controlled by Presentation 14.9 (Neurobehavioral Systems) using a Windows computer (HP Z200 SFF, Hewlett-Packard Company) and a 19-inch CRT monitor (HM903D-A, Iiyama) with a refresh rate of 150 Hz and a resolution of 1024 × 768 pixels. A response box (RB-530, Cedrus) was used for the visual search task. Before the experiment began, participants performed 20 practice trials of the visual search task to familiarize themselves with the apparatus.

### Procedure

#### Visual search task

Participants sat in a chair with their chin fixed in a steady position 80 cm from the monitor. They were asked to focus their gaze on a fixation cross (0.9° × 0.9°) at the center of the display when the cross was presented.

A total of 432 trials were presented in six blocks of 72 trials, with an equal number of target-present and target-absent trials. Each target condition appeared 54 times in a pseudo-randomized order. In each trial, the fixation cross was presented for 500 ms, followed by a stimulus array of four faces presented until the participant responded (Fig 1B). Participants were asked to respond as quickly and accurately as possible by pushing the appropriate button on a response box using their left or right index finger to indicate whether all four faces were the same or one face was different. The position of the response buttons was counterbalanced across participants.

#### Rating

After the visual search task, the participants performed the rating task for individually-presented facial stimuli. Participants were asked to evaluate each stimulus in terms of intensity of emotional arousal and nature of emotional valence that they felt while perceiving the stimulus expression using a nine-point scale measuring from 1 (“low arousal” and “negative valence”) to 9 (“high arousal” and “positive valence”). To assess the possible confounding factors, participants also rated familiarity (i.e., frequency with which the facial expression is encountered in daily life) and naturalness (i.e., degree to which the expression seemed natural) using a nine-point scale from 1 (“not at all”) to 9 (“very much”). The order of the presentation of the stimuli and rating items was randomized and balanced across participants.

### Data analysis

#### Visual search task

All statistical tests were performed using IBM SPSS Statistics 22 (IBM Corporation). Statistical significance was set at *p* < .05. The mean RTs of correct responses in target present trials were calculated for each condition after excluding artifacts (measurements greater than ±3*SD*). A three-way repeated-measure analysis of variance (ANOVA) was used to compare RTs, with neuroticism (high/low) as a between-participants factor and stimulus type (normal/anti-expression) and emotion (anger/happiness) as within-participant factors. Follow-up analyses were conducted using simple-effect tests for significant interactions; main effects or lower-order interactions were not subject to interpretation when higher-order interactions were present [25].

Accuracy was high for all conditions in both the high- and low-neuroticism groups (mean ±*SE* %; normal-anger 94.8 ± 1.1, 93.5 ± 1.8; normal-happiness 94.1 ± 1.2, 91.7 ± 1.7; anti-anger 85.6 ± 2.6, 83.2 ± 3.1; anti-happiness 86.8 ± 2.2, 83.3 ± 3.3 for the high- and low-neuroticism groups, respectively). Preliminary analyses showed no evidence of the effects of neuroticism on accuracy, *F*(1, 36) < 0.83, *p* > .36. Therefore, we report only the RT results for detecting facial targets.

#### Rating

Ratings of arousal, valence, familiarity and naturalness were analyzed in the same manner as RTs.

#### Rating-RT relationship

A multiple regression analysis was performed on participants with high and low levels of neuroticism. To test the between-response variability (vs. the between-participant variability; [26]), the dependent variable was the mean RT for each participant under each condition (i.e., normal-anger, normal-happiness, anti-anger, and anti-happiness), independent variables were the rating scores (effects of interest). Dummy variables were used to represent participants (effects of no-interest). The interaction between neuroticism (high/low) and rating (i.e., arousal, valence, familiarity, and naturalness) was tested to investigate the slope difference in the regression lines between the high- and low-neuroticism groups.

## Results

### RT

**Fig 2:**
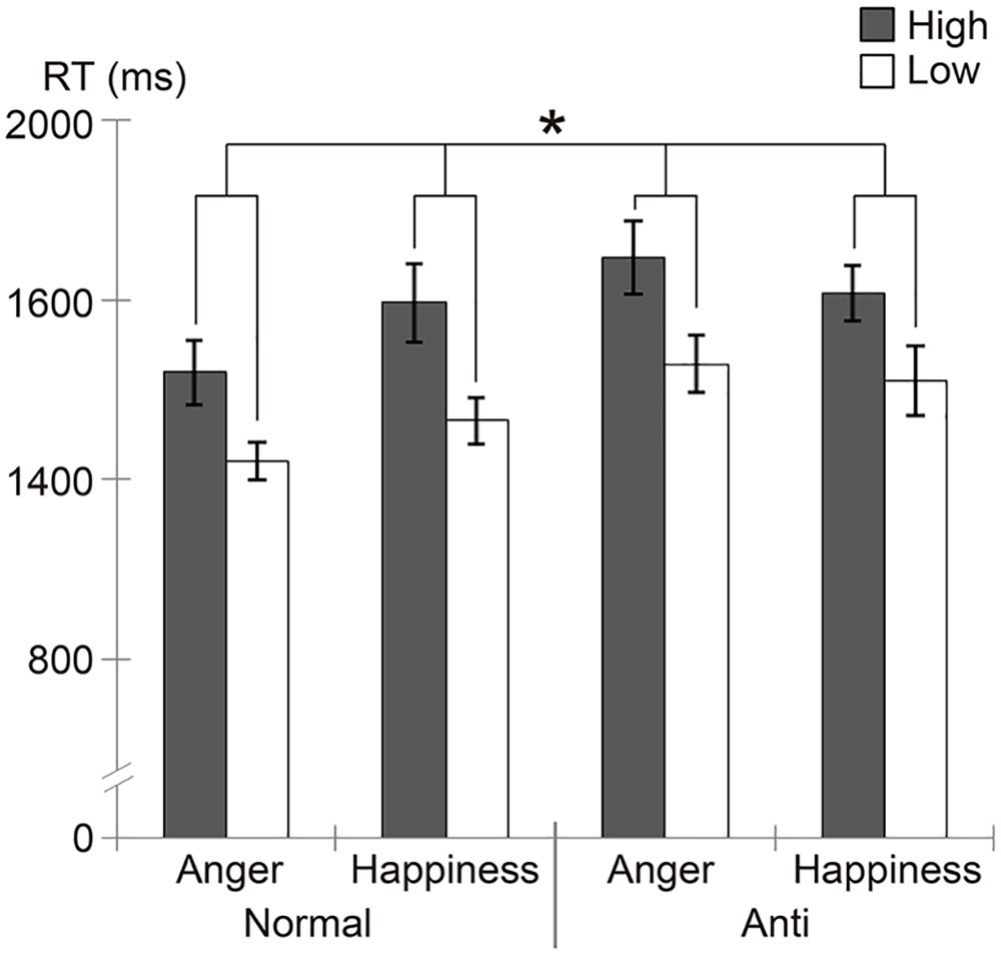
Reaction time (RT; mean ± *SE*) for each target condition in the visual search task in participants with high- and low-neuroticism scores. Significant main effects of neuroticism (*p* < .05) are indicated with an asterisk.

RTs for each target condition are shown in Fig 2. We performed a repeated-measure ANOVA with neuroticism, stimulus type, and emotion as factors. The results showed a significant main effect of neuroticism, *F*(1, 36) = 6.43, *p* = .02, *η*_*p*_^*2*^ = .15, indicating that the high-neuroticism group was slower to detect facial expression targets than the low-neuroticism group. No interaction effects related to neuroticism were found, *F*(1, 36) < 2.31, *p* > .10.

Additionally, the analysis revealed a significant main effect of stimulus type, *F*(1, 36) = 40.19, *p* < .001, *η*_*p*_^*2*^ = .53, and a significant interaction between stimulus type and emotion, *F*(1, 36) = 13.39, *p* = .001, *η*_*p*_^*2*^ = .27. In the follow-up analyses for the interaction, a significant simple-effect of stimulus type was found for anger, *F*(1, 72) = 68.11, *p* < .001, *η*_*p*_^*2*^ = .65, indicating that the normal expression of anger was detected faster than its corresponding anti-expression. The results also showed a significant simple-effect of emotion for normal expressions, *F*(1, 72) = 33.01, *p* < .001, *η*_*p*_^*2*^ = .48, indicating that normal-anger was detected faster than normal-happiness.

### Rating

Ratings of each item are shown in Table 1. For arousal, a three-way ANOVA with neuroticism, stimulus type, and emotion as factors revealed a significant main effect of neuroticism, *F*(1, 36) = 7.09, *p* = .01, *η*_*p*_^*2*^ = .17, indicating that high-neuroticism participants gave higher emotional arousal ratings to facial expressions than low-neuroticism participants did. In the other rating items, there were no significant effects or interactions related to the neuroticism factor, *F*(1, 36) < 3.13, *p* > .09.

**Table 1:** Subjective ratings of arousal, valence, familiarity, and naturalness (mean ± *SE*) in the high- and low-neuroticism groups.

(a) Arousal				
	Normal		Anti	
Neuroticism	Anger	Happiness	Anger	Happiness
High	7.1 (0.3)*	6.4 (0.2) *	5.1 (0.3) *	5.1 (0.2) *
Low	6.6 (0.3)	5.9 (0.3)	4.6 (0.2)	3.6 (0.3)
(b) Valence				
	Normal		Anti	
Neuroticism	Anger	Happiness	Anger	Happiness
High	2.8 (0.2)	7.2 (0.3)	5.0 (0.2)	4.2 (0.3)
Low	2.9 (0.2)	6.5 (0.3)	4.8 (0.3)	3.7 (0.2)
(c) Familiarity				
	Normal		Anti	
Neuroticism	Anger	Happiness	Anger	Happiness
High	3.8 (0.4)	7.3 (0.2)	4.6 (0.3)	3.7 (0.3)
Low	3.3 (0.3)	7.2 (0.3)	4.5 (0.4)	4.0 (0.3)
(d) Naturalness				
	Normal		Anti	
Neuroticism	Anger	Happiness	Anger	Happiness
High	4.7 (0.6)	7.4 (0.3)	5.4 (0.3)	4.7 (0.4)
Low	4.8 (0.4)	7.0 (0.3)	5.8 (0.4)	5.1 (0.4)

* Significant main effect of neuroticism (*p* < .05).

### Rating-RT relationship

#### Arousal-RT relationship

**Fig 3:**
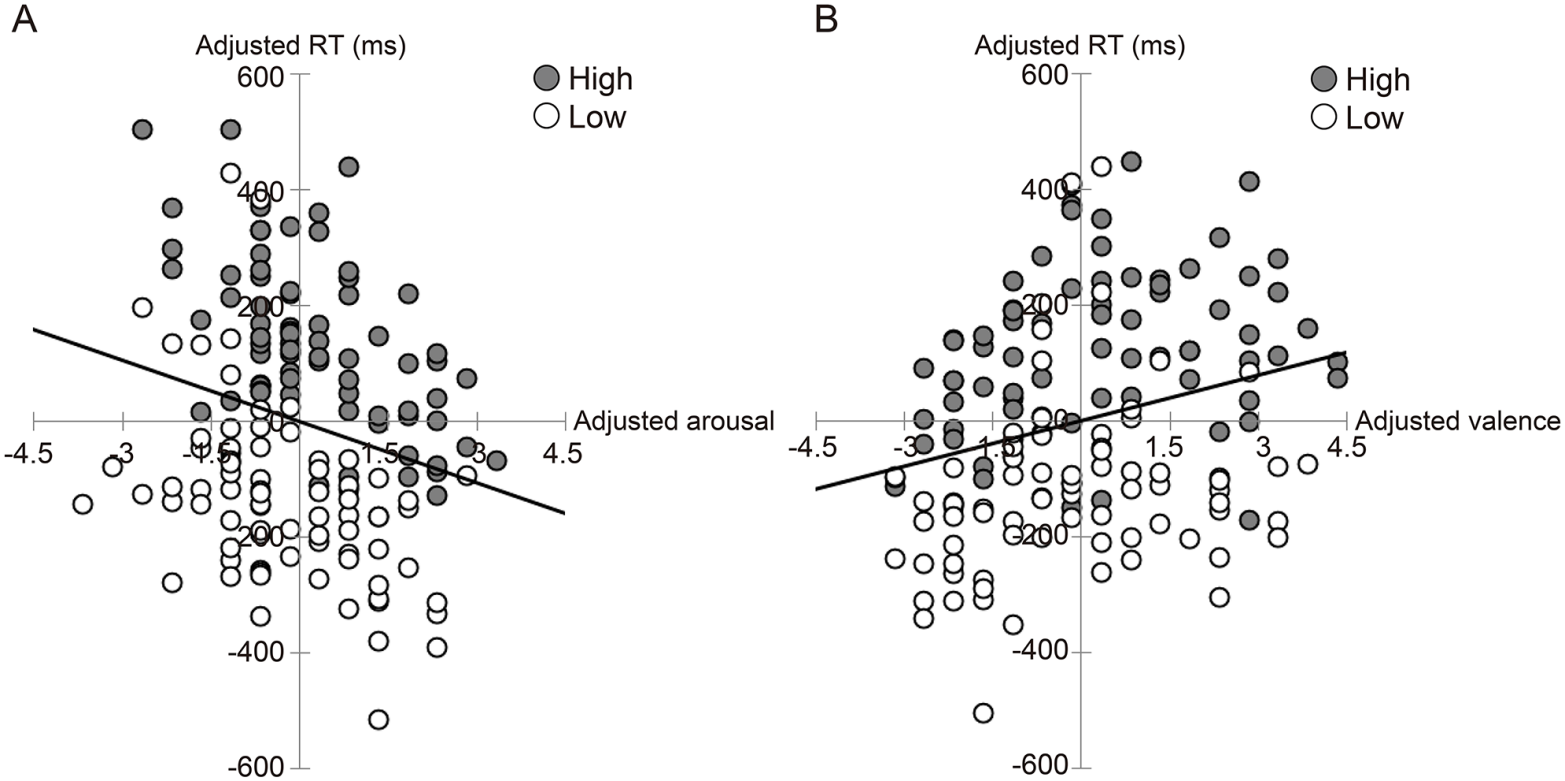
The relationship between emotional arousal and RT (A) and between emotional valence and RT (B). Gray circles and white circles represent data from the high- and low-neuroticism groups, respectively. Scatter plots and regression lines represent the relationships between the ratings and RTs after partialling out the participant effect and subtracting the means of the RTs and ratings.

The analyses showed that the interaction between neuroticism and arousal rating was not significant, *F*(1, 112) = 0.36, *p* > .10, indicating no significant slope difference in the regression lines of the high- and low-neuroticism groups. The analyses showed a significant relationship between emotional arousal and RTs, *F*(1, 112) = 29.44, *p* < .001. These results indicate that higher arousal ratings were associated with more rapid facial expression detection for both high- and low-neuroticism participants (Fig 3A).

#### Valence-RT relationship

The analysis revealed that the interaction between neuroticism and valence rating was not significant, *F*(1, 112) = 0.14, *p* > .10, indicating There was no significant slope difference between the regression lines of the high- and low-neuroticism groups. The analyses did show a significant relationship between emotional valence and RTs, *F*(1, 112) = 7.18, *p* < .01. These results demonstrate that negative valence was associated with shorter RTs for detecting target facial expressions (Fig 3B).

#### Non-emotional rating-RT relationship

The regression analysis showed no interaction between neuroticism and familiarity, *F*(1, 112) = 0.02, *p* > .10, or between neuroticism and naturalness ratings, *F*(1, 112) = 0.46, *p* > .10. Additionally, significant relationships were not found between familiarity and RT or between naturalness and RT, *F*(1,122) < 1.00, *p* > .10.

## Discussion

To investigate the modulatory effect of neuroticism on rapid detection of emotional facial expressions, we asked participants to perform a visual search task in which those with high and low scores of neuroticism detected normal expressions of anger and happiness, as well as their anti-expressions, within crowds of neutral expressions in photographs. We also collected subjective emotional ratings in response to facial expressions.

The general patterns of RTs across the high- and low-neuroticism groups indicated that normal expressions of anger were detected more rapidly than the corresponding anti-expressions. Normal angry expressions were also detected more rapidly than normal happy expressions. Regression analyses showed a negative relationship between arousal rating and RT, suggesting that a higher degree of emotional arousal facilitates the rapid detection of facial expressions. The analyses also showed a positive relationship between valence rating and RT, indicating that more negative feelings toward facial expressions are more rapidly detected. These results are consistent with those of previous studies [14, 16, 17]. Healthy participants with both high and low levels of neuroticism detected emotional faces, especially angry expressions, more rapidly than neutral faces. Their enhanced emotional arousal or their negative feelings facilitated the detection of facial expressions.

More important, our results showed the modulatory effects of neuroticism on the detection of facial targets. First, participants with high scores of neuroticism were slower to detect facial expressions than participants with low scores of neuroticism, regardless of stimulus type and emotion. This result is consistent with a previous study that showed that high-neuroticism participants had a delay in detecting facial expressions during a similar visual search task, although the researchers did not compare emotional and neutral facial targets [15]. Second, subjective ratings of arousal for facial expressions were higher in the high-neuroticism group than in the low-neuroticism group, indicating that participants with high levels of neuroticism experience an enhanced intensity of emotional feelings in response to facial expressions. This is consistent with a previous study that reported that high-neuroticism participants showed more emotional responses to a given situation when they received negative feedback on their tasks [10]. Based on these two results, participants with high-neuroticism showed a delay in detecting facial expressions compared to participants with low-neuroticism, although high-neuroticism participants experienced higher arousal in response to these facial expressions than did low-neuroticism participants. Our findings are in line with previous studies showing that high-neuroticism participants were less accurate in their recognition of emotional facial expressions [7], or had less gaze maintenance to the eye regions of facial expressions of others [8, 9] compared to low-neuroticism participants. Taken together, it is suggested that people who show high levels of neuroticism avoid emotional signals in facial expressions to protect themselves from possible, but unfounded, worries [6].

Contrary to our expectations, there was no significant slope difference in the valence-RT relationship between the high- and low-neuroticism groups. This null finding means that negative feelings in response to facial expressions facilitate rapid detection in healthy participants who are either high or low in neuroticism. However, when we preliminarily performed separate regression analyses for the high- and low-neuroticism groups, the results revealed a significantly positive relationship between emotional valence and RT in the high-neuroticism group, *t*(56) = 2.55, *p* = .01, whereas this relationship did not reach significance in the low-neuroticism group, *t*(56) = 1.42, *p* > .10. Considering previous studies that reported that high-neuroticism participants responded more acutely than low-neuroticism participants to emotional signals of negative facial expressions [11], future studies should test larger samples to determine whether more neurotic individuals have more volatile tendencies based on perceived threat levels [6].

Our results have implications for the modulatory effects of neuroticism on social interaction via emotional facial expressions. Neuroticism can be one of the best predictors of the risk of depression [5]. Some studies have reported that, compared to healthy individuals, individuals with depression showed a more pronounced tendency to avoid emotional facial expressions [27]. Although our study focused exclusively on healthy adults, the current results suggest that avoidance or overestimation of emotional arousal in response to emotional facial expressions may lead to problems in communicating via facial expressions and result in social withdrawal in depressed patients.

The results can also offer insights regarding the rapid processing of facial expressions in the visual search paradigm. Several studies have found that the detection of emotional facial expressions is more rapid than the detection of neutral expressions [13]; however, it is not clear if this can be attributed to emotional or visual processing [28]. Previous studies evaluating emotional experience and detection RT have found that rapid detection of emotional facial expressions can be attributed to emotional significance rather than visual factors [14]. This is supported by our finding that neuroticism, which is strongly related to emotional vulnerability, modulated the detection of facial expressions and subjective emotional arousal in response to facial expressions. It is suggested that the rapid detection of facial expressions is attributable to the emotional significance of the facial expressions.

This study had some limitations. First, we used only two emotional expressions (i.e., anger and happiness). As the primary purpose of our study was to investigate the effect of neuroticism in detecting facial expressions, both negative (angry) and positive (happy) affects are considered to be effective. However, some researchers have found differing effects of neuroticism on the recognition of facial expressions based on the category of emotion [29]. Therefore, future studies should utilize additional categories of emotional expressions to assess differences in emotional information processing between high- and low-neuroticism groups.

Second, we measured the five-factors of personality and did not investigate facets of neuroticism, such as anxiety. Because several studies have reported a modulatory influence of anxiety on emotional expression recognition [11], it is possible that anxiety accounted for the effect of neuroticism that we found in this study. Although a previous study reported a null effect of the trait anxiety on the detection of the target emotional expression in the visual search paradigm [30], it is difficult to come to a conclusion, due to several differences in methodologies across studies. Investigation of facets of neuroticism with respect to the effect on facial expression detection would be an important consideration for future research.

Third, all participants were Japanese and all stimuli were photographs of Caucasian faces. Although several studies have shown that emotional expression processing is largely universal across cultures [31], some studies have reported that cultural bias exists in decoding emotional facial expressions [32]. It is possible that behavioral manifestations of neuroticism modulate such cultural biases in the detection of emotional facial expressions. Future studies including participants and stimuli from multiple cultures should assess cultural biases in the relationship between personality and facial expression detection.

In conclusion, our data provide evidence that neuroticism decreased the speed of the overall detection of facial expressions and enhanced emotional arousal in response to facial expressions. Such withdrawal from facial signals can allow people with high levels of neuroticism to keep themselves away from possible, but unfounded, worries.

## Supporting Information

S1 Dataset Datasets of visual search task and ratings of the high- and low-neuroticism participants. (XLS)

S1 Table Big-five scores of the high- and low-neuroticism groups. (DOC)
