# Supplementary material for: Neuroticism Delays Detection of Facial Expressions
Source: PLoS One. 2016 Apr 13;11(4):e0153400. doi: 10.1371/journal.pone.0153400 (PMC4830574; doi:10.1371/journal.pone.0153400)
Supplement: S1 Table — (DOC) [file pone.0153400.s002.doc]

*S1 Table 1. Big five scores (mean ± SE) of the high- and low-*neuroticism groups.

|  | Neuroticism | |
| --- | --- | --- |
|  | High | Low |
| Neuroticism | 38.6 (0.7) | 19.8 (1.1) |
| Extraversion | 22.9 (2.1) | 28.5 (1.6) |
| Openness | 29.4 (1.1) | 34.6 (1.1) |
| Agreeableness | 29.7 (1.0) | 31.1 (1.5) |
| Conscientiousness | 22.5 (2.0) | 26.0 (1.9) |
